# Case report: Emerging therapies for transformed small cell lung cancer: efficacy of serplulimab and a comprehensive case report

**DOI:** 10.3389/fmed.2024.1406515

**Published:** 2024-09-25

**Authors:** Heng-Xu Lyu, Wen-Hua Ma, Yong-Qian Zhang, Hui Jin, Yu-Dong Wang, Min Zhao

**Affiliations:** ^1^Department of Oncology, The First Hospital of Hebei Medical University, Shijiazhuang, China; ^2^Department of Medical Oncology, The Fourth Hospital of Hebei Medical University, Shijiazhuang, China

**Keywords:** transformed small cell lung cancer (T-SCLC), immune checkpoint inhibitors (ICIs), immunotherapy, non-small cell lung cancer (NSCLC), case report

## Abstract

This research reports a case of histological transformation from non-small cell lung cancer (NSCLC) to transformed small cell lung cancer (T-SCLC) in a patient undergoing EGFR-tyrosine kinase inhibitors (TKIs). The aggressive characteristics of the tumor diverged significantly from those commonly associated with lung adenocarcinomas, leading to further histological analysis. The subsequent histological examination confirmed the transformation to SCLC, consistent with established mechanisms of acquired resistance in NSCLC. Given the limited therapeutic options, the patient was administered a serplulimab-based immunochemotherapy regimen, achieving a progression-free survival (PFS) of 6 months post-transformation. The study underscores the potential of PD-1 inhibitors, particularly serplulimab, in the treatment landscape for T-SCLC and highlights the need for future comprehensive research.

## Introduction

Lung cancer is traditionally classified into two main histological categories: small-cell lung cancer (SCLC) and non-small cell lung cancer (NSCLC), with SCLC comprising approximately 15% of cases (1, 2). Recognized for its aggressive nature and tendency for metastasis, SCLC presents a notably poor prognosis, reflected in a 5-year survival rate of < 7% (2–4). In recent years, oncologists have observed a phenomenon known as lineage plasticity, particularly prevalent in the field of lung cancer (5). Specifically, NSCLC may transform into SCLC following treatments such as tyrosine kinase inhibitors (TKIs), chemotherapy, or immunotherapy, and approximately 15% of epidermal growth factor receptor (EGFR)-mutant lung adenocarcinomas (LUAD) undergo histological transformation to SCLC following acquired resistance to TKIs (6, 7).

Transformed small cell lung cancer (T-SCLC) shares a similarly poor prognosis with conventional SCLC, reflecting one of the most aggressive and lethal forms of lung cancer (8). Standardized treatment strategies are notably absent for T-SCLC, and patients are managed with platinum-etoposide (PE) chemotherapy predominantly (8, 9). Previous studies have reported a median overall survival (OS) of only 6 to 10 months and a median progression-free survival (PFS) of 3 or 4 months following a diagnosis of T-SCLC (8, 10, 11). The comparably short survival and therapeutic dilemmas highlight the complexity of managing T-SCLC and emphasize the urgent need for more effective therapeutic strategies.

The IMpower 133 studies have demonstrated the efficacy of adding the programmed death-ligand 1 (PD-L1) inhibitor atezolizumab to the PE regimen (12, 13). Compared to chemotherapy alone, this combination has improved OS and PFS in patients with extensive-stage SCLC (ES-SCLC), establishing it as a first-line treatment option for ES-SCLC. Furthermore, the ASTRUM-005 also verified the survival benefit of programmed cell death protein 1 (PD-1) inhibitor serplulimab combined with etoposide and carboplatin in ES-SCLC (14). Recently, the National Medical Products Administration (NMPA) approved a marketing application for a new indication of anti-PD-1 monoclonal antibody drug toripalimab injection in combination with etoposide and platinum for the first-line treatment of ES-SCLC, which provides a new option for the treatment of ES-SCLC. These have prompted a revolutionary breakthrough in the immunochemotherapy of SCLC for over 30 years. In a retrospective study, the incorporation of atezolizumab and chemotherapy for T-SCLC patients has been found to indicate a trend toward extending PFS and OS (15). These findings provide a promising clinical insight, suggesting that immune therapies targeting the PD-L1/PD-1 axis, proven effective in SCLC, may also hold potential for treating T-SCLC.

This report details the case of a patient who transformed into SCLC from EGFR TKI-resistant LUAD. Initially diagnosed with EGFR-mutated LUAD, the patient was treated with the third-generation TKI, osimertinib, followed by anlotinib as a second-line therapy. After transformation to SCLC at month 14 after first-line treatment, the patient was administered a combination therapy of the PD-1 inhibitor serplulimab, along with etoposide and carboplatin, achieving a PFS of 6 months.

## Case presentation

### Initial management of lung adenocarcinoma

**Figure 1:**
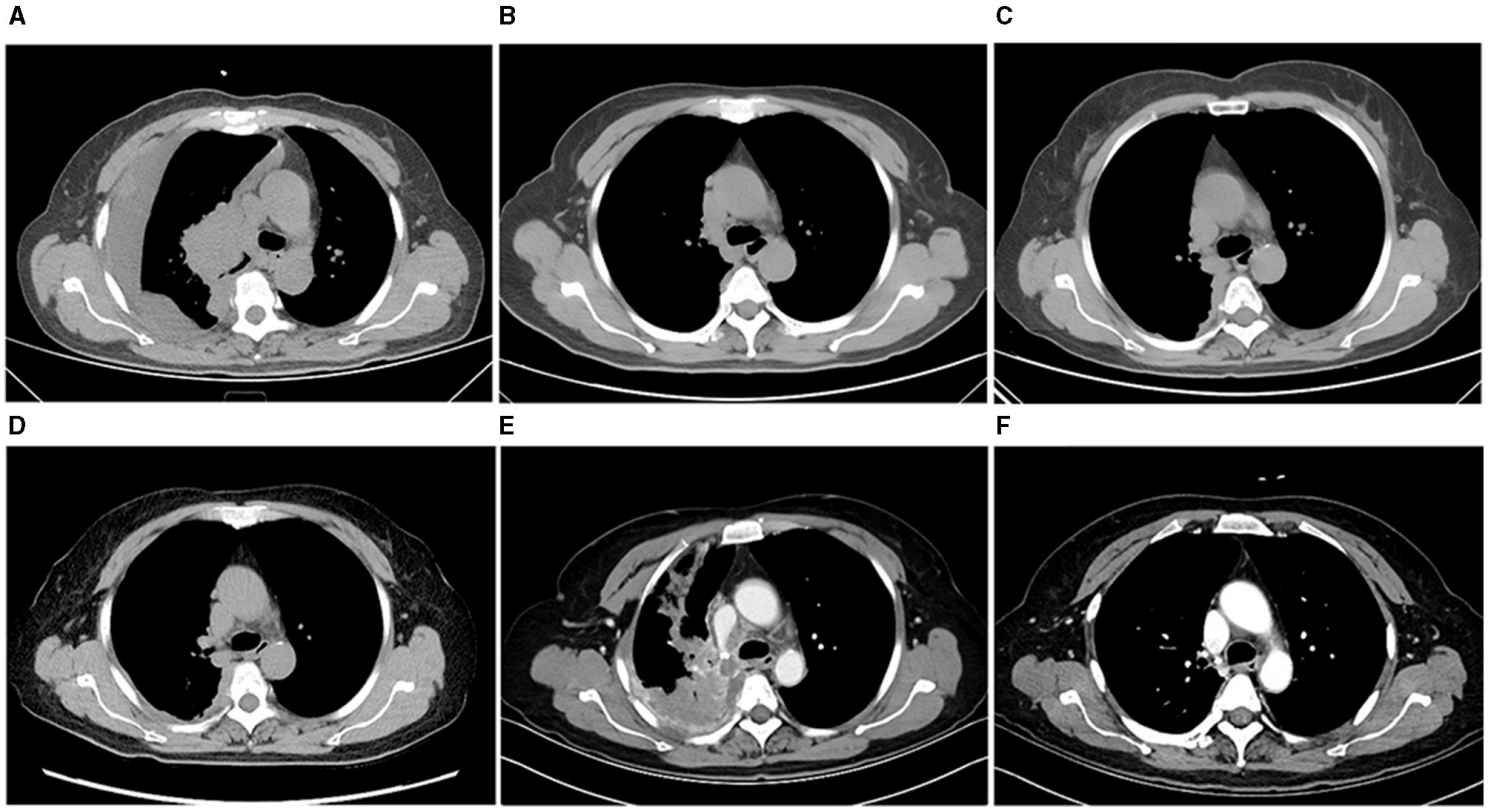
Evolution of thoracic tumor as revealed by chest CT imaging. **(A)** CT image at the time of initial diagnosis. **(B)** Partial response (PR) after first-line treatment. **(C)** Disease progression (PD) after first-line treatment. **(D)** Disease stable (SD) after second-line treatment. **(E)** PD after histological SCLC transformation. **(F)** PR after serplulimab-based treatment.

**Figure 2:**
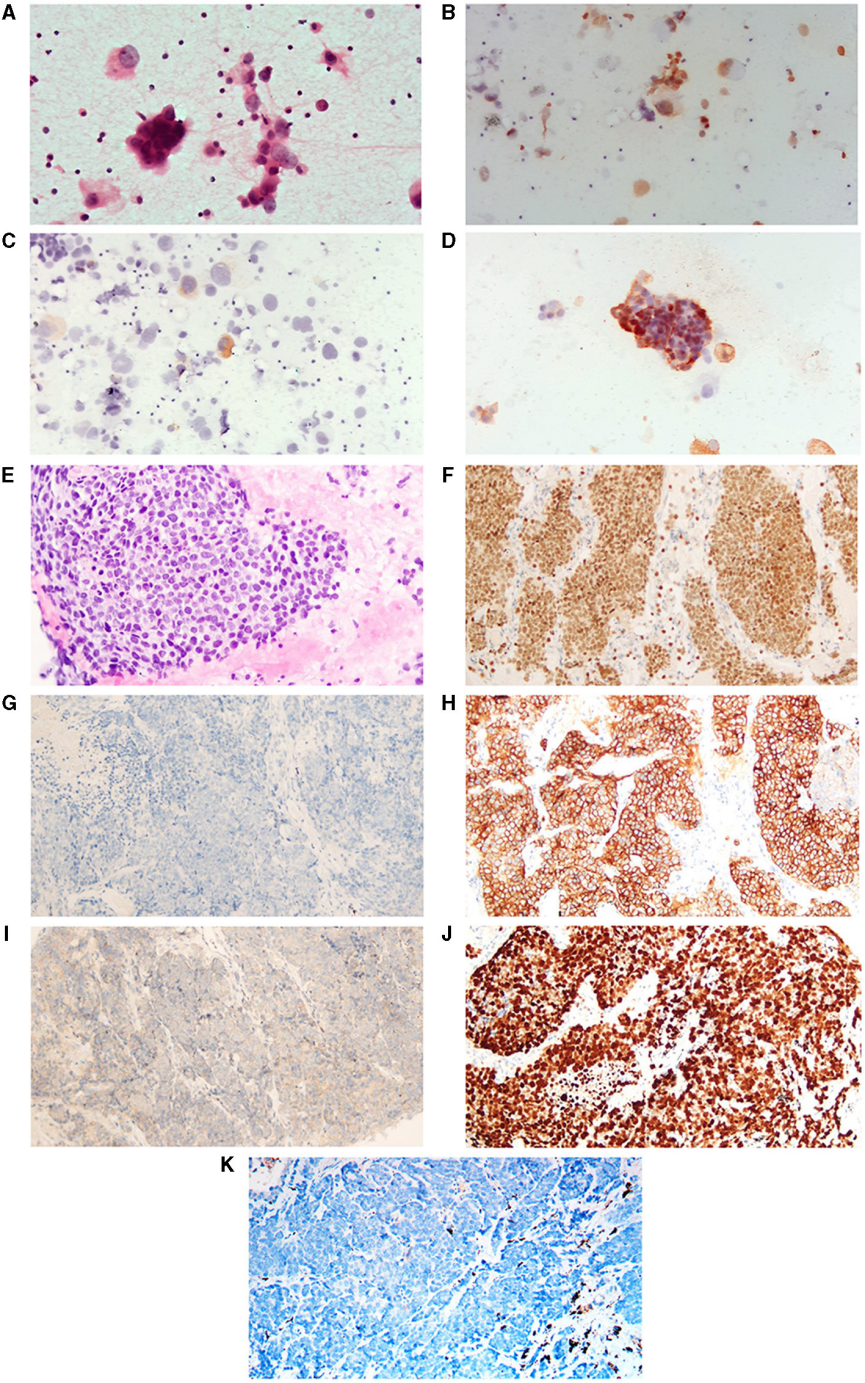
H&E and immunohistochemistry staining of tumor. **(A–D)** Represent cytologic photographs of lung adenocarcinoma (LAUD, before the histological transformation of the tumor). **(A)** Displays a hematoxylin and eosin (H&E) stained aspirate cytology sample. **(B–D)** Are positive immunohistochemical staining photographs for thyroid transcription factor-1 (TTF-1), Napsin A, and carcinoembryonic antigen (CEA), respectively. **(E–K)** Are pathological pictures following the SCLC transformation. **(E)** Shows H&E staining. **(F–K)** Are immunohistochemical images demonstrating positivity for TTF-1, negativity for Napsin A, and positivity for Synaptophysin (Syn), CD56, Ki-67, and negativity for programmed death-ligand 1 (PD-L1), respectively. All images are presented at 400x.

A 65-year-old female patient presented to the Fourth Hospital of Hebei Medical University in September 2021 with symptoms of cough, sputum production, and dyspnea. She reported no smoking or any notable medical history; general physical examination and routine laboratory tests showed no abnormalities. Chest CT imaging demonstrated a mass at the right hilum accompanied by obstructive pneumonitis, nodularities on the right pleural surface, a pleural effusion on the right leading to lung atelectasis, and prominent mediastinal lymph nodes (Figure 1A). Cytologic analysis from the thoracentesis identified an abundance of atypical cells. Immunocytochemical staining revealed positive markers for TTF1, CK7, CEA, and NapsinA, but negative for WT-1, CDX2, GATA3, and PD-L1 (22C3) (Figures 2A–D). Next-generation sequencing (NGS) identified an *EGFR* L858R mutation in exon 21 with a frequency of 68.0%, and a *PTEN* mutation abundance of 10.2%, with no mutations detected in *RB1* and *TP53* genes. Based on the radiographic findings combined with the cytologic evaluation from the pleural fluid, the patient was diagnosed with stage IV lung adenocarcinoma (T2N2M1) with an Eastern Cooperative Oncology Group (ECOG) performance status (PS) of 1.

Following the confirmed diagnosis and in line with clinical guidelines, the patient was initiated on osimertinib (80 mg daily, orally), complemented by concurrent intrapleural cisplatin hyperthermic perfusion therapy (120 mg, every 10 days for four cycles). The best objective response observed was partial response (PR) based on RECIST criteria, with a PFS of 9 months (Figure 1B). By August 2022, a PET/CT scan was conducted due to shoulder pain, which displayed further disease progression, signified by metastatic involvement in the right pleura, mediastinum, right hilar lymph nodes, right iliac bone, and the right second posterior rib. Upon observing disease progression, anlotinib was administered orally at a dosage of 12 mg daily for a cycle of 14 consecutive days, followed by a 7-day discontinuation as a second-line treatment (Figures 1C, D). However, an echocardiographic assessment in September 2022 revealed a decline in the left ventricular ejection fraction (LVEF) to 38%, indicating a potential cardiomyopathy linked to TKI therapy. As a result, anlotinib treatment was discontinued.

### Histological transformation to SCLC and subsequent management

In November 2022, the patients came to the First Hospital of Hebei Medical University for further treatment. Following first-line osimertinib therapy, the patient demonstrated continuous and widespread disease progression. The tumor's biological behavior began to diverge from the typical characteristics of lung adenocarcinoma. Given literature reports suggesting that histological transformation could be one of the mechanisms underlying tumor drug resistance, a repeat tumor biopsy was performed in November 2022. Pathological analysis identified the presence of poorly differentiated carcinoma. The subsequent immunohistochemical findings were as follows: TTF-1(+), CK7(–), NapsinA(–), Syn(+), CgA(–), CD56(+), Ki-67(>90%+), CK5/6(–), P40(–), P63(–), PD-L1(–), and MOC31(+) (Figures 2E–K). NGS revealed an *EGFR* L858R mutation in exon 21 with a frequency of 29.93%, and a *PTEN* mutation abundance of 86.32%, with no mutations detected in *RB1* and *TP53* genes. Additionally, the LVEF measured in November 2022 was 41%. Based on these results, the patient was diagnosed with extensive-stage small cell lung cancer, with an ECOG PS of 3.

There's no consensus in the medical community on a definitive treatment strategy for patients with T-SCLC. Historically, treatment predominantly revolved around etoposide combined with platinum-based chemotherapy (cisplatin/carboplatin), but these regimens offered limited efficacy. Reports indicate that combining immunotherapy and platinum-based chemotherapy can extend the PFS for T-SCLC patients compared to chemotherapy alone (12, 13). Moreover, the ASTRUM-005 study demonstrated that the combination of serplulimab, etoposide, and carboplatin as first-line therapy significantly prolonged PFS and OS for patients with ES-SCLC. Based on this emerging evidence, our patient was prescribed a regimen of serplulimab (200 mg on day 1), etoposide (70 mg/m^2^ from days 1–5), and carboplatin (AUC 5 on day 1), administered in 6-week cycles. The patient achieved a PR by the end of the second cycle in January 2023, and this response was maintained even after five cycles of the combination therapy, concluding in March 2023 (Figures 1E, F). The patient's general condition was alleviated rapidly following treatment. Alongside supportive care, there was a notable improvement in the LVEF, which normalized by the end of the second cycle. Additionally, the ECOG PS was evaluated as 1. However, the patient declined subsequent maintenance therapy with serplulimab.

**Figure 3:**
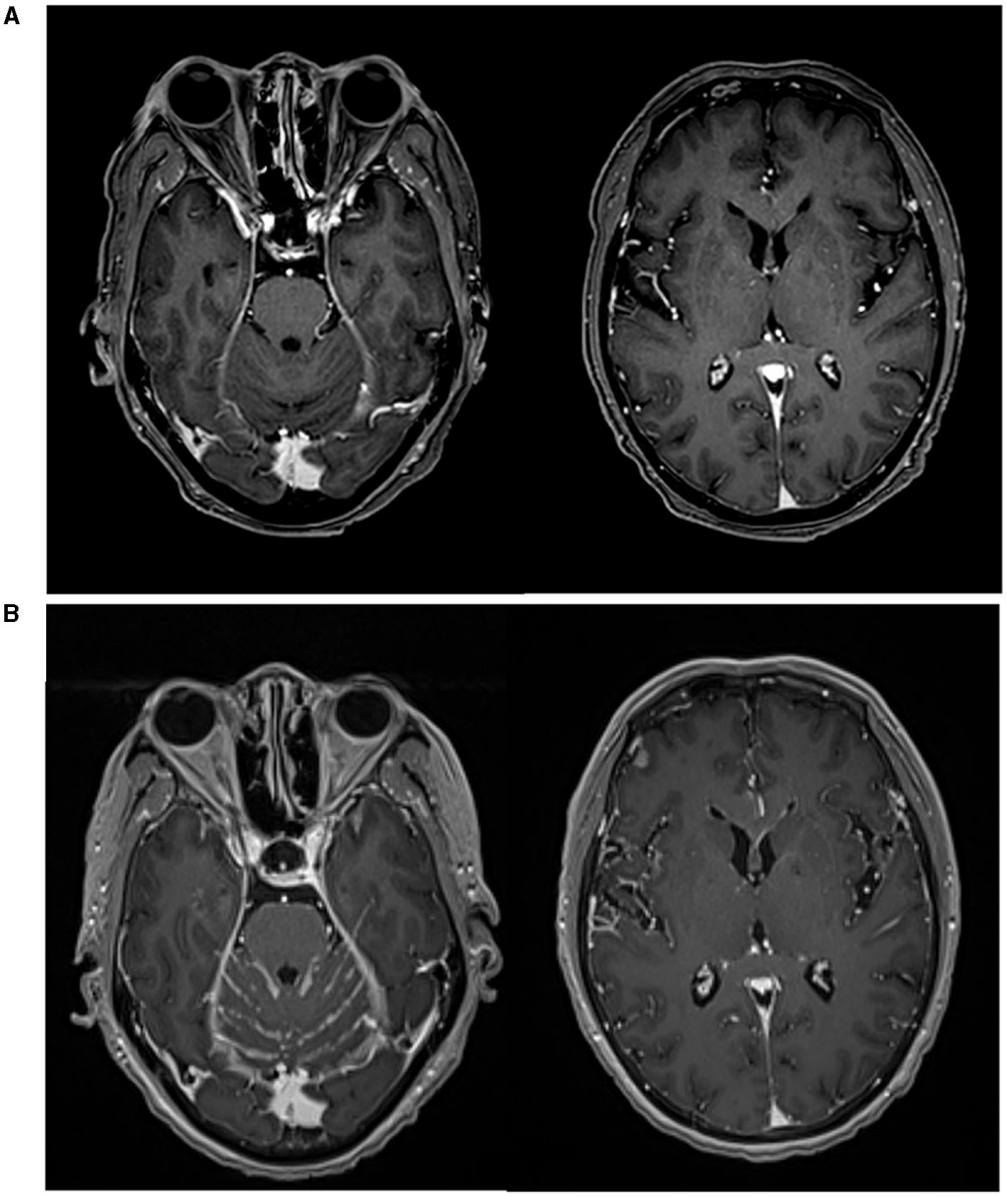
Brain MRI scans. **(A)** MRI image at the time of SCLC transformation. **(B)** MRI confirmed metastasis to the central nervous system (CNS).

In May 2023, the patient was readmitted presenting with symptoms of nausea and vomiting. Comprehensive diagnostic evaluations revealed that the pulmonary lesions remained stable. However, brain MRI and lumbar puncture conclusively identified metastasis to the central nervous system (CNS) (Figure 3). Unfortunately, localized radiation therapy yielded a suboptimal response. After a multidisciplinary team review, it was projected that her life expectancy is under 12 weeks, especially considering her deteriorated general health status reflected by an ECOG Performance Status of 4. Subsequently, the patient and her family chose to forego further treatment, opting for hospice care. The patient passed away later in the same month, marking an OS of 20 months since her lung cancer diagnosis.

## Discussion

Acquired resistance following EGFR-TKI therapy is recognized as one of the mechanisms underlying the transformation from NSCLC to SCLC (6), and the transformation can only be conclusively diagnosed through histological methods. In the case we reported, the patient's PFS with the first-line osimertinib treatment was 9 months, significantly shorter than the previously reported 18.9 months (16). Moreover, the subsequent treatment with anlotinib yielded limited efficacy. After these two lines of systemic therapy, the patient's tumor exhibited a marked change in biological behavior, displaying increased aggressiveness, growth rate, and speed of metastasis, more akin to SCLC than the conventional lung adenocarcinomas typically associated with NSCLC. Coupled with literature evidence that histological transformation is one of the mechanisms of acquired resistance in treated NSCLC (especially in lung adenocarcinomas) (6, 17), these clues led us to suspect a potential histological transformation in this patient, prompting confirmation through a repeat biopsy. The patient's histological profile confirmed SCLC, originating from lung adenocarcinomas. Studies have generally demonstrated that histological transformation to SCLC occurs 16–19 months following treatment with EGFR-TKIs (5, 7, 8, 11). In contrast, the patient in our report experienced this transformation in the 14^th^ month after osimertinib first-line treatment, slightly earlier than the literature.

Research into the complex molecular processes governing the transformation from NSCLC to SCLC has illuminated potential therapeutic pathways. Genomic sequencing revealed that the T-SCLC tumor tissue retains the *EGFR* mutation, although preclinical and clinical evidence indicates resistance to *EGFR* inhibition (18, 19). This feature is also observed in our case, where the transformed tumor still harbors the *EGFR* mutation, albeit at a reduced abundance. Through histological analysis, we found that the transformed tumor tissue exhibits morphological and neuroendocrine characteristics identical to those of classical SCLC, which are consistent with previous research (7). The medical consensus generally attributes the histological transformation to the concomitant biallelic loss of *TP53* and *RB1* (7). Further studies have shown that T-SCLC patients are frequently characterized by molecular features mirroring those of classical SCLC, including the presence of an activating mutation in *PIK3CA*, loss of heterozygosity, loss of *RB1*, and an inactivating mutation in *TP53* (7, 20, 21). In our case, the patient did not exhibit mutations in *TP53* and *RB1*; however, a histological transformation still occurred. Indeed, while SCLC is characterized by cells that are functionally mutant for RB and p53 proteins, not all SCLC cases demonstrate inactivation of the *RB1* and *TP53* genes (22). It is important to consider that the undetected mutations in *RB1* and *TP53* in our patient could be attributed to the spatial heterogeneity of the tumor microenvironment (23), and hence, we cannot exclude the presence of alterations in *TP53* and *RB1*. In summary, the observed similarities in tumor histology and molecular characteristics between T-SCLC and classical SCLC indicate that therapeutic strategies for classical SCLC may also apply to patients with T-SCLC.

Notably, in this case, the patient experienced a substantial increase in the mutation abundance of PTEN (from 10.2% to 86.32%) following histological transformation. This observation suggests the potential involvement of the PI3K pathway in the patient's histological transition. Furthermore, the upregulation of the PI3K-AKT signaling pathway has been substantiated in this transition, as evidenced by EGFR-mutant patient-derived xenograft models where inhibition of the PI3K/AKT pathway arrested tumor growth and neuroendocrine transformation (24). Additional studies have illuminated the complexity of the transformation, revealing the downregulation of NOTCH signaling and overexpression of MYC and BCL2 (5, 24, 25). These studies also offer potential therapeutic strategies for patients with T-SCLC. Notably, the immune microenvironment between LUAD and T-SCLC exhibits distinct differences, particularly marked by downregulation of immune modulators and a conspicuous decline in CD8+ T cell presence in T-SCLC (26), highlighting the potential role of immunotherapy in managing this transformation. A multicentre retrospective study reported by Fujimoto et al. showed that after 15 SCLC patients were treated with PD-1/PD-L1 inhibitor monotherapy, only 1 patient was adequate, with an mPFS of 1.3 months (27). ICIs alone have limited efficacy in transformed SCLC. Therefore, in our case, following the histological transformation of the patient, we initiated a serplulimab-based immunochemotherapy regimen, achieving a PFS of 6 months, which is superior to the previously reported PFS of < 4 months (11).

Cardiotoxicity is a common adverse reaction to TKIs such as osimertinib. A study has indicated that cardiac adverse events (AEs) occur in approximately 5% of patients with EGFR-Mutated NSCLC treated with osimertinib (28). Although clinical research suggests that TKIs like osimertinib may increase the risk of cardiotoxicity, including heart failure, no evidence indicates that TKIs have direct treatment-related cardiotoxicity (29, 30). In this case, the patient experienced a severe reduction in LVEF and a significant decline in general condition following TKIs therapies. We consider that the patient's rapid tumor growth, following resistance to osimertinib and anlotinib, increased pulmonary burden, which in turn led to myocardial ischemia, thus triggering cardiotoxicity. Consequently, there was no notable improvement after discontinuation of the drug, which ultimately accelerated disease progression. The low LVEF state also posed challenges to the patient's safety medication. After a multidisciplinary consultation and considering the excellent safety profile of serplulimab in conjunction with the patient's preference, it was decided to administer serplulimab combined with EP chemotherapy for the treatment of T-SCLC, against the backdrop of supportive therapy to improve cardiac ejection function. The patient tolerated the serplulimab-based treatment well, rapidly improving the ECOG PS score and restoring cardiac function to normal levels.

For T-SCLC patients, recent research has begun to explore beyond traditional PE chemotherapy, seeking innovative solutions that reflect the complexity of the disease. In a retrospective study of 47 T-SCLC patients, incorporating atezolizumab into the existing chemotherapy treatment demonstrated a promising shift toward longer PFS (from 4.1 to 5.1 months) and substantially enhanced OS, increasing it from 7.9 to 20.2 months (15). Further corroborating these findings, a specific case of SCLC transformation from lung adenocarcinoma has shown a durable response to durvalumab (a PD-1 inhibitor) and PE regimen up to 19 months (31), reinforcing the potential efficiency of PD-1/PD-L1 inhibitors combined with chemotherapy for transformed SCLC. This initial evidence has been pivotal in motivating further investigations into immunochemotherapy as a viable alternative. Serplulimab, a novel PD-1 targeting monoclonal antibody developed by Shanghai Henlius Biotech, Inc., has emerged as a promising contender. Acting to reinvigorate the immune system's ability to recognize and attack tumor cells, serplulimab has shown encouraging results in trials such as ASTRUM-005 (NCT04063163) (14). The clinical success of serplulimab, achieving a PFS of 6 months in our case, extends beyond previous retrospective studies, laying the groundwork for a potential paradigm shift in treating T-SCLC.

Upon examining *ClinicalTrials*, we identified two prospective studies focused on T-SCLC. The first is an investigator-initiated, open-label, prospective phase II clinical trial, identified by NCT05957510, set to be conducted across various centers in China, aiming to enroll 36 patients with T-SCLC who have not received prior treatment after undergoing histological transformation, with the primary objective of evaluating the efficacy and safety of serplulimab in combination with chemotherapy in the treatment of EGFR-mutated NSCLC transformed into SCLC after treatment with safety and efficacy (32). The second, known by NCT04538378 in the United States, is designed to enroll 14 subjects with EGFR-mutated T-SCLC, having undergone transformation following EGFR-TKI and having been treated with platinum-based chemotherapy. While there are slight variations in the designs of these two studies, both aim to assess the antitumor activity and safety of immunochemotherapy regimens containing PD-1/PD-L1 inhibitors for T-SCLC patients.

In conclusion, this case report illustrates a promising response to serplulimab in a T-SCLC patient, providing a glimpse into the potential efficacy of incorporating PD-1 inhibitors with traditional chemotherapy. The complex landscape of T-SCLC, encompassing its early detection, underlying mechanisms, and clinical management, remains a fertile ground for exploration and discovery. Further comprehensive research, ranging from basic pre-clinical studies to retrospective and prospective clinical trials, is paramount to illuminating this unique subtype of lung cancer.

## Data Availability

The original contributions presented in the study are included in the article/supplementary material, further inquiries can be directed to the corresponding authors.
